# Process evaluation of an effective Antimicrobial Stewardship intervention in hospitalized patients with community-acquired pneumonia

**DOI:** 10.1017/ash.2026.10340

**Published:** 2026-03-30

**Authors:** I. van Heijl, S.E.J.D. van den Eijnde, V.A. Schweitzer, C.H. van Werkhoven, M.E.J.L. Hulscher, P.D. van der Linden, M.J.M. Bonten

**Affiliations:** 1 Department of Pharmacy, Rijnstate Hospital, Arnhem, Netherlands; 2 Julius Centre for Health Sciences and Primary Care, University Medical Centre Utrecht, Utrecht, Netherlands; 3 Department of Pharmacy, Tergooi Medical Centre, Hilversum, Netherlands; 4 IQ Health Science Department (IQ Health), Radboud University Medical Center, Nijmegen, Netherlands; 5 European Clinical Research Alliance on Infectious Diseases (Ecraid), Utrecht, Netherlands

## Abstract

**Objective:**

In a previously conducted stepped-wedge cluster randomized trial, a multicomponent Antimicrobial Stewardship intervention reduced mean duration of broad-spectrum antimicrobial treatment by 26.6% (from 6.5 to 4.8 days) in patients with moderate-severe community-acquired pneumonia (CAP). This study presents a detailed process evaluation of the multicomponent Antimicrobial Stewardship improvement intervention.

**Methods:**

The intervention consisted of educational activities (clinical lessons with prescription feedback, e-learning, pocket cards, and poster), local opinion leaders promoting guideline-adherent treatment, and prospective audit and feedback. The experiences with performing the intervention were evaluated using trial data; exposure data (clinical lessons, e-learning, and prospective audit and feedback); and semi-structured interviews conducted in December 2019 with eleven physicians from four hospitals.

**Results:**

Most intervention components were conducted as planned. However, clinical lessons and e-learning were scheduled more frequently, while prospective audit and feedback was temporarily interrupted in some hospitals. Clinical lessons were on average attended by 31% of the target audience and 44% completed the e-learning. Feedback was provided in 56% of patients receiving broad-spectrum antimicrobials. Clinical lessons, prospective audit and feedback, and local opinion leaders, were identified as the most effective components. Pocket cards were regarded as easy to implement. While e-learning content was judged useful, its perceived effectiveness was reduced by “e-learning fatigue.” The poster was considered of low effectiveness.

**Conclusion:**

Based on participant experiences, key components of an effective stewardship intervention for improving antimicrobial use in hospitalized CAP patients included prospective audit and feedback, clinical lessons with prescription feedback, pocket cards, and actively engaged local opinion leaders.

## Introduction

Community-acquired pneumonia (CAP) is an acute lower respiratory tract infection associated with high mortality and morbidity.^1,2^ In the Netherlands, the recommended empirical therapy for patients with moderate-severe CAP is amoxicillin or penicillin.^3^ However, adherence to the national CAP guideline in clinical practice is suboptimal, and many patients are unnecessarily treated with broad-spectrum antimicrobials.^4–6^ Unnecessary use of antimicrobials contributes to the emergence of resistance, increases healthcare costs, and elevates risk of side effects.^7–9^

Reducing excessive use of broad-spectrum antimicrobials is a major focus of Antimicrobial Stewardship programs (ASPs) globally. ASPs aim to minimize inappropriate antimicrobial therapy while optimizing outcomes.^10^ In general, stewardship strategies can effectively increase guideline adherence, resulting in less broad-spectrum antimicrobial use, shorter treatment durations, and a reduction of adverse drug reactions.^11^ To decrease the overuse of broad-spectrum antimicrobials in patients with moderate-severe CAP, we conducted an investigator-initiated, stepped-wedge clustered-randomized, non-inferiority, Antimicrobial Stewardship trial.^12^ This trial evaluated a multicomponent ASP intervention targeting healthcare providers and demonstrated a 26.6% (95%CI: 18.0–35.3%) relative reduction in days of therapy (DOT) with broad-spectrum antimicrobials, with non-inferiority regarding 90-day mortality. However, there was considerable heterogeneity between hospitals, with reductions in prescribing ranging from 16.7% to 39.3%.

Process evaluations are essential for understanding study outcomes and evaluating multicomponent interventions. They help determine which elements of an intervention are most effective and support the improvement of interventions.^13^ Process evaluations of hospital-based ASPs evaluated in clinical trials are rarely performed.^14^ Also, most published studies on ASP focus on reporting outcomes, but lack a detailed description of their intervention components. This process evaluation aims to present a comprehensive description of the multicomponent ASP intervention, evaluate the relative contribution of its components, and provide practical recommendations for implementing ASPs in hospitals where appropriate antimicrobial use for patients with moderate-severe CAP is needed.

## Methods

### Study design

This process evaluation is performed as part of a previously conducted stepped-wedge cluster-randomized trial in nine Dutch hospitals (CAP-PACT trial).^12^ Between November 2015 and November 2017, 2,235 patients were included during the control period and 1,849 during the intervention period. The multicomponent ASP intervention aimed to improve guideline adherence for antimicrobial treatment of moderate-severe CAP. It was based on previously established improvement interventions proven to be effective in hospitals.^15^ The intervention consisted of A) educational activities (clinical lessons with prescription feedback, e-learning, pocket cards, and poster), B) local opinion leaders, and C) prospective audit and feedback on patient-level antimicrobial use (Supplement S1–S5). Local opinion leaders from internal medicine, pulmonology, and medical microbiology were identified for their perceived influence on prescribing behavior and tasked with promoting guideline adherence during the intervention. Each hospital appointed an opinion leader as principal investigator (PI) and had an established Antimicrobial Stewardship Team before study initiation. However, no CAP-specific stewardship activities were conducted. The target group for the intervention were physicians (both specialists and residents) from the departments of pulmonary and internal medicine. Each intervention component was classified according to the Effective Practice and Organisation of Care Taxonomy for Implementation strategies, category: interventions targeted at health care workers (Supplement S6).^16^

### Process evaluation framework

The process evaluation was based on a framework that describes the key features of an improvement intervention, and aimed to explore participant’s exposure to, experiences with, and suggestions for improving the intervention components (Supplement S7).^17^ For each component, where possible, the intervention as planned, observed exposure to the intervention, and recommendations to improve the intervention were assessed (Table 1).

**Table 1.** Process evaluation framework

Process evaluation component	Measure	Data source
Intervention as planned	Description of intervention as defined in study protocol and carried out during study period	Trial data (Table 3, Supplement S1–S5)
Observed exposure to the intervention	How the physicians were exposed to the intervention	Exposure data, trial data (Table 2, Supplement S8)
Recommendations to improve the intervention	How the physicians experienced the intervention	Semi-structured interviews, participation data (Table 4, Supplement S9–S10)

### Data collection

Different data sources were used (Table 1):

1. Trial data containing the study protocol and study data were used to describe the intervention as planned.
2. Hospital participation rates in clinical lessons, e-learning, and performance data from prospective audit and feedback was used to describe exposure to the intervention components. Exposure to pocket cards, poster and opinion leaders could, for pragmatic reasons, not be measured.
3. Experiences of participants to the first clinical lesson was used to assess quality, practical use, knowledge acquisition and suggestions for improvement.
4. Semi-structured interviews were conducted with opinion leaders involved in and exposed to the multicomponent intervention to assess their overall experiences and to collect suggestions for improvements. Participants were asked which (set of) components they considered most or least effective in reducing broad-spectrum antimicrobial use. Additionally, a six-point scoring system, ranging from 1 (bad) to 6 (excellent), was used to assess the overall appraisal of the individual components. To facilitate accurate assessment, the poster was shown during interviews if not initially recalled by participants. Interviews were voice-recorded and transcribed verbatim. Four hospitals were purposively selected based on their stewardship results to maximize variation (Table 2). Hospital 1, with moderate baseline performance, yielded a moderate intervention effect. Hospital 2, initially the best performing hospital, had a moderate reduction in broad-spectrum antimicrobials. Hospital 3, starting at a moderate level, demonstrated a large reduction. Hospital 4, the lowest performer initially, achieved a substantial reduction in broad-spectrum antimicrobials. At the time of the interviews, eight hospitals still actively carried out the ASP intervention, seven as part of a follow-up study (Clinicaltrials.gov NCT03360851) and one independently. All opinion leaders were invited to participate via email. Initially, two interviews were planned per hospital, each involving one or two participants. Additional interviews were scheduled until data saturation was achieved, as judged by the researchers (IH, PL). The interviews were conducted in December 2019, two years after the primary end point data collection for the CAP-PACT trial. This delay resulted from the study’s extensive scale, prolonged data processing, and the subsequent recognition of the added value of qualitative insights. The interviewer used an interview guide structured around the framework, covering the aim, discussion topics, and core questions.

**Table 2.** Characteristics, observed exposure and effects of hospitals participating in interviews

Hospital	Characteristics (No. of hospital beds)	% e-learning completion	% clinical lesson attendance	% feedback acceptance rate	% adjusted effect^a^	Median DOT (control period)	Median DOT (intervention period)	% adherence^b^ (control period)	% adherence^b^ (intervention period)
1	Teaching hospital (500–750)	25.5	52.7	100	21.9	4	3	39.1	49.5
2	Teaching hospital (500–750)	52.6	30.7	73.7	16.7	2	0	59.2	69.1
3	General hospital (<500)	40.0	46.7	88.9	28.4	7	2	26.7	63.6
4	Teaching hospital (500–750)	72.1	32.3	41.8	28.6	8	5	14.7	27.3
Total		43.7	31.0	59.7	26.6	6	3	28.8	45.9

a Effect was measured as the average decrease in days of therapy with broad-spectrum antimicrobials (DOT) adjusted for confounders (PSI-score, smoking status, COPD, diabetes mellitus, and antimicrobial pretreatment).

b Guideline adherent antimicrobial therapy: narrow-spectrum antimicrobials as empirical therapy (penicillin, amoxicillin, or doxycycline).

### Data analysis

The first three data sources were analyzed descriptively, using means and standard deviations (SD) to summarize experiences related to the first clinical lesson. Transcripts were analyzed using the thematic content approach, which involves systematically identifying and interpreting patterns of themes within the data. Data were collected and analyzed concurrently, allowing emerging themes to be incorporated and explored in subsequent interviews. The transcripts were read in full, after which the data were coded based on the key features using NVIVO 12.0. One researcher (IH) assigned codes. To reduce inter-subjectivity in the coding process, a second researcher (PL) verified whether the assigned codes adequately covered all relevant data. Disagreements were solved by discussion.

## Results

### Trial data to describe the intervention as planned

Most interventions were conducted as initially planned (Table 3). However, clinical lessons and e-learning modules were scheduled more frequently than initially intended. Prospective audits were conducted once daily, with feedback usually provided one day after empirical therapy initiation. In some hospitals, brief periods without prospective audit and feedback occurred due to temporary staff unavailability.

**Table 3.** Intervention as planned

Intervention component	Intervention as planned
Clinical lessons	The lessons were initially planned every six months, but were scheduled every 3–4 months to reach missed or newly employed physicians while avoiding irritation. A total of 54 lessons were conducted by coordinating researchers across 9 hospitals, with a mean interval of 108 days (3.9 months) between lessons. The first lessons included a study introduction, relevant theory, case-based discussions on the national CAP guideline, and anonymized prescriber performance comparisons across hospitals (Supplement S1). Attendance was recorded, and feedback forms were distributed to evaluate the lessons, using a scoring system ranging from 1 (bad) to 6 (excellent). Follow-up lessons consisted of a brief theory review, prescription data feedback, and anonymized prescriber performance comparisons across hospitals. Preparation time was approximately three hours, and lessons were scheduled three months in advance, mostly during preexisting educational time slots. The first lesson in each hospital lasted approximately 30 minutes, with follow-up lessons lasted 15 minutes (retrospectively estimated by coordinating researchers).
E-learning	E-learnings were sent every 3–4 months to all participants and monthly to new employees, including theoretical content and case-based questions related to the national CAP guideline. Developed using Google Forms, the e-learning included eight case-based questions (Supplement S2) and was comparable to the clinical lessons, serving as a booster. Coordinating researchers emailed the PIs the e-learning link, requesting forwarding to the target group. The link was sent two weeks after the first clinical lesson, with follow-up emails to confirm it had been sent. Estimated completion time for the e-learning was approximately ten minutes. Response rates were monitored by coordinating researchers, and at least once during the intervention, each PI received an update with a list of completed e-learnings and a request to follow up with non-responders.
Pocket card	Pocket cards were distributed to the target group at the start of the intervention by local opinion leaders, during the first clinical lesson, as part of the standard onboarding protocol for new medical staff, and later by the local study team upon request. Pocket cards summarized main recommendations for empirical CAP treatment and were adapted locally in collaboration with PIs and Antimicrobial Stewardship teams. Developed in Word, the cards were printed with a plastic coating in A5 format to fit in a coat pocket (Supplement S3). Pockets cards were distributed to either specialists and residents or residents only, depending on local preferences.
Poster	The poster was distributed to the target group at the start of the intervention period, containing a concise overview of the CAP guideline. At the start of the intervention period, pocket cards were printed based on the estimated number of current and new employees over at least one year. Additional pocket cards were printed on request. Local study teams were requested to display the poster in physicians’ offices located within the emergency department, pulmonology ward, and internal medicine ward. The poster was developed in PowerPoint in A4 format and printed in color on thick white glossy paper (Supplement S4).
Engaging opinion leaders	Local opinion leaders from internal medicine, pulmonology and medical microbiology were identified in collaboration with local Antimicrobial Stewardship teams prior to the start of the intervention period, based on their perceived influence on prescribing behavior during routine clinical practice. They were tasked with promoting guideline adherence throughout the intervention. Initially, meetings with local opinion leaders were planned every six months to discuss past performances and barriers to guideline adherence. However, these discussions were instead integrated into clinical lessons, where opinion leaders were usually present.
Prospective audit and feedback	Prospective audit and feedback were provided by local Antimicrobial Stewardship teams (Supplement S5). For auditing, admission charts were reviewed on weekdays to identify patients with moderate-severe CAP. For feedback, physicians were contacted by telephone and advised to switch to penicillin or amoxicillin monotherapy if treatment deviated from guideline recommendations or if deviations were not appropriately motivated in the medical records. If a switch was not possible, physicians were recommended to perform a pneumococcal urine antigen test to facilitate de-escalation if the result was positive. Feedback was documented in electronic health records. Audit was conducted once daily, often in the morning. As a result, feedback was usually provided one day after empirical therapy initiation. Audit and feedback were scheduled on all weekdays, however, in some hospitals short periods without feedback occurred due to illness or holidays without available replacement staff.

CAP, community-acquired pneumonia; PI, principal investigator.

### Participation data to describe exposure

The total target group consisted of 520 physicians: 218 specialists and 302 residents. Average attendance per clinical lesson was 31% of the target audience (range: 11%–53%). Over the entire study period, 227 individuals (44%; range: 2.1%–72%) completed the e-learning module. Completion rates were 47% among residents and 39% among specialists, with seven participants completing the e-learning twice. Feedback from daily audits was provided for 330 of the 591 patients (56%) who received broad-spectrum antimicrobials (Supplement S8).

### Experience data to assess quality and suggestions for improvement of clinical lessons

After the first clinical lessons, 227 feedback forms were completed (Supplement S9 and S10). The mean (±SD) scores, out of a maximum of 6, were 4.4 (±0.7) for lesson quality, 4.5 (±0.9) for practical use, and 4.1 (±0.9) for knowledge acquisition. Participants perceived the lessons as useful, clear, and interesting. Additionally, the prescription data feedback and case-based discussions were well appreciated. Suggestions for improvement included adding more case-based questions and modifying open-ended case-based questions to multiple-choice questions.

### Semi-structured interviews to describe experiences and recommendations

Initially, nine opinion leaders participated in eight interviews. In two hospitals, two opinion leaders were not involved in all intervention components. To address this, a pulmonology resident who received the intervention was interviewed for additional insights, as well as a medical microbiology resident who had only provided prospective feedback. The two participants solely involved in feedback were interviewed about this component only. Interviews lasted an average of 33 minutes (range: 10–53 min).

Table 4 summarizes the participants’ experiences and recommendations for improvement. Overall, clinical lessons, prospective audit and feedback, and local opinion leaders were identified as the most effective components, while the e-learning and poster were considered the least effective. Clinical lessons, pocket cards, and prospective audit and feedback received the highest overall appraisal scores. The target groups for clinical lessons and e-learning were generally considered appropriate, although inclusion of emergency physicians initiating empirical CAP therapy was recommended to ensure representation of all relevant medical specialties. Both prescription data feedback and face-to-face contact during the clinical lessons were highly valued. Pocket cards were considered easy to use and preferred over a smartphone application, which would require additional resources. It was noted that local opinion leaders were not equally active in promoting guideline adherence within hospitals. Accordingly, increased efforts were recommended to ensure equal engagement by opinion leaders from all departments involved in treatment of CAP patients. Finally, inclusion of a pop-up in the electronic patient files, providing immediate feedback upon prescribing broad-spectrum antimicrobials was recommended to reduce unnecessary use.

**Table 4.** Experiences and recommendations for improvement of the ASP intervention components (acquired during interviews)

Intervention components	Experiences	Recommendations for improvement
(A) Educational activities		
Clinical lessons	Mean perceived overall appraisal score: 5.3 (SD: 0.4) Target group was appropriately selected. Duration and frequency of the lessons were adequate during the study period. The content conveyed a clear message. Theoretic information was perceived as too easy for specialists. Aggregated feedback on hospital-level prescribing performance, including comparison across hospitals, was the most appreciated part and it motivated to perform better. Face-to-face contact was well appreciated. Facilitators: (1) scheduling lessons within a fixed educational program and (2) planning them well in advance. Barriers: clinical duties and night or weekend shifts prevented attendance.	Invite all medical specialties involved in treating patients with CAP. Provide an appealing announcement beforehand, including the lesson topic. (eg presentation of results). Supervisors should encourage residents to attend. Ensure that presenter is knowledgeable about CAP and has access to prescription data to demonstrate prescription performance. Reduce frequency of lessons to once per year after implementation.
E-learning	Mean perceived overall appraisal score: 4.3 (SD: 1.3) Target group was appropriately selected. Duration of the e-learning, its content and the medium itself were adequate. Barriers: a full e-mail inbox, low prioritization, the voluntary aspect, e-learning “fatigue.”	Invite all medical specialties involved in treating patients with CAP. Add accreditation or make completion mandatory. Complete the e-learning in groups during clinical lessons. Share response rates more frequently with target group. Send invitations through a supervisor or a specialist from a different department.
Pocket card	Mean perceived overall appraisal score: 5.1 (SD: 0.6) Target group was appropriately selected. Format and content were adequate. Medium was well appreciated. Use varied per hospital: either by residents only or by both residents and specialists. Facilitators: easy in use, minimal effort required to grab card from a pocket.	Develop a webpage compatible with mobile phones or a digitally accessible PDF document, although pocket cards remain preferred due to their quicker accessibility.
Poster	Mean perceived overall appraisal score: 3.7 (SD: 1.6) Poster was not remembered by any participant. Location and size were adequate. Content was unclear. The added value of a poster as an intervention in clinical practice was questioned. Barriers: no designated person was responsible for maintaining or updating the poster in the emergency department.	Inquire upfront whether a hospital prefers to use a poster. Supply on plastic-coated paper to enhance durability. Revise content and lay-out to improve clarity.
(B) Engaging opinion leaders	Mean perceived overall appraisal score: 3.8 (SD: 1.0) Opinion leaders were not equally active in promoting guideline adherence. Selected persons were naturally active opinion leaders; being selected as opinion leader they became more active. Activity of opinion leaders was most pronounced during hand-over meetings. Barriers: policy from another (academic) hospital limited guideline adherence due to perceived pressure to avoid narrow-spectrum antimicrobials.	Have multiple active opinion leaders, ideally representing each medically involved specialty. Organize a start meeting with all local opinion leaders and hold regular follow-up meetings to discuss past performance and barriers that impede adherence.
(C) Prospective audit and feedback	Mean perceived overall appraisal score: 5.3 (SD: 0.8) Overall, the feedback was well received by physicians and in good harmony. Few occasions led to frustrating reactions, but these were always resolved through discussion. Feedback was provided to physicians on the ward currently caring for the patient, while often the physician on duty in the emergency department had prescribed empirical therapy the previous day. Some feedback providers did not register the feedback in the electronic patient file and provided it only by telephone. Over time, justification for deviation from the recommended empirical treatment was better recorded in patient files. Some supervisors disagreed with the feedback and stopped the residents from narrowing antimicrobial therapy. Most of the time was spent on the audit component: approximately 60 minutes on Mondays and 15–30 minutes from Tuesday to Friday.^a^	Specialists, including supervisors and local opinion leaders, should agree with the policy to treat according to guideline. Register feedback in electronic patient file so that it can be accessed and reviewed by the next physician on duty. Implement an algorithm (clinical rule) to identify CAP patients, ensuring continuity of the audit and feedback process and reducing time required for auditing.

SD, standard deviation; CAP, community-acquired pneumonia.

a Based on data from one hospital.

### Comparative evaluation of intervention across hospitals

In hospital 1, three participants (opinion leader pulmonology, feedback provider from the medical microbiology department, and intervention receiver) were interviewed. Initially, prospective audits were conducted by medical microbiologists. However, due to the time required, a research nurse took over, with only complex cases reviewed and feedback provided by the medical microbiologist. One participant identified clinical lessons with prescription feedback as the most effective component, while another viewed both clinical lessons and activation of opinion leaders as the most effective components. Mean attendance for clinical lessons was 52.7%, and feedback acceptance rate was 100% (Table 2). One participant noted that the opinion leader in their department actively promoted high-quality care, although this had also been the case prior to the study, while another opinion leader was less engaged. The feedback provider reported smooth communication with residents and specialists. Finally, the e-learning and poster were regarded as the least effective components, particularly in clinical practice.

In hospital 2, both participants (opinion leaders internal medicine and medical microbiology department) valued clinical lessons and prospective audit and feedback the most effective components, while the poster was identified least effective. They regarded each other as highly active opinion leaders, with one identifying this as one of the most effective components. E-learning completion was 52.6%, and feedback acceptance rate was 73.7% (Table 2). To support prospective audit and feedback, the hospital had implemented an automated daily algorithm to identify patients with presumed CAP receiving non-guideline adherent antimicrobials. As a result, this component remained actively implemented at the time of the interview, independent of study participation.

In hospital 3, all participants (opinion leaders pulmonology, internal medicine and medical microbiology) regarded clinical lessons the most effective component. Two participants identified prospective audit and feedback as equally effective, while one considered opinion leaders as equally effective as clinical lessons. Clinical lessons attendance averaged 46.7%, and feedback acceptance rate was 88.9% (Table 2). The small number of physicians resulted in direct communication and less variation in opinions. Two participants viewed the e-learning, and one the poster, as the least effective components.

In hospital 4, three participants were interviewed (opinion leaders pulmonology and medical microbiology department, and feedback provider). A research nurse conducted prospective audit and referred relevant cases to the feedback provider. One participant identified clinical lessons and providing feedback equally effective, while another valued prospective audit and feedback as the most effective. Participants reported pressure from medical specialists at a nearby academic hospital, whose policy differed from the national guideline, to deviate from the guideline. Overall, feedback acceptance rate was 41.8% (Table 2). One participant regarded the e-learning, and another the poster, as least effective components.

## Discussion

This study provides a process evaluation of a multicomponent Antimicrobial Stewardship intervention that successfully influenced physicians’ prescribing behavior, leading to a 26.6% relative reduction in DOT with broad-spectrum antimicrobials in hospitalized CAP patients. Prospective audit and feedback, clinical lessons, and engagement of local opinion leaders were considered the most effective intervention components, while the e-learning and poster were perceived the least effective and, therefore, potentially redundant components.

Opinion leaders, clinical lessons and prospective audit and feedback have been identified as key components for influencing prescribing behavior.^11,15,18,19^ However, the opinion leader component in this study was perceived as only moderately effective. To improve this, interviewees recommended selecting opinion leaders from each relevant specialty, with regular meetings to evaluate performance and address barriers to guideline adherence. All participants identified clinical lessons among the most effective components, despite only 31% of the target audience attending the first lesson. Based on our findings we recommend lessons to be scheduled during existing educational sessions, include all relevant medical specialties, and supervisors actively encouraging residents to attend. Prospective audit and feedback were considered both effective and well appreciated by the participants, though it required 15 to 60 minutes per day. The use of algorithms to select patients for feedback was recommended, as this could reduce time required for audit. Pocket cards were not identified as either the most or least effective component, and were scored comparable for overall appraisal as clinical lessons, but were considered easy to implement. Their use varied across hospitals, with residents using them in teaching hospitals, and both residents and specialists in the general hospital. The e-learning content was appreciated, but due to an overload of competing e-learning invitations, it received lower scores compared to clinical lessons. Finally, none of the interviewees recalled the poster, and it remained unclear whether, or for how long, it was displayed.

Detailed reporting of performed Antimicrobial Stewardship interventions and conducting process evaluations are essential for enabling reproducibility and facilitating comparison of outcomes across studies.^11,14,20^ Generalizability of ASP studies largely depends on the quality of implementation of the different intervention components. Additionally, qualitative studies focusing on the barriers and facilitators of ASP components are needed to address gaps in understanding factors that influence prescribing behavior and to identify the most effective strategies for future ASP efforts.^21,22^ Although the intervention study was conducted between 2015 and 2017, this process evaluation conducted in 2019 provides valuable insights into how the components were implemented, how they were received by stakeholders, and how they might be improved. Despite technological advances, adherence to CAP guidelines remains suboptimal,^23–25^ and our findings remain relevant to effective Antimicrobial Stewardship.

The multicomponent ASP intervention was applied as a one-size-fits-all approach, but reductions in broad-spectrum antimicrobial DOT varied across hospitals. Previous studies have suggested that locally adapted ASPs may be more effective than standardized approaches.^14,26^ Designing the intervention as a toolkit and allowing hospitals to tailor components to their local context, could enhance its effectiveness. For example, although the e-learning content was considered valuable, it was perceived as one of the least effective components. In hospitals already offering numerous e-learning opportunities, and where low engagement with the module was anticipated, this component could reasonably have been omitted with efforts redirected towards more effective components.

A known barrier to implementing prospective audit and feedback is its time-consuming nature.^27^ Although not explicitly mentioned in the interviews, possibly because some interviewees were no longer directly involved in the audit, prior meetings with PIs and researchers suggest that the time required for auditing was negatively perceived. One hospital introduced an algorithm to automate auditing and continued to carry out prospective audit and feedback outside of study participation. Automating the identification of CAP patients with broad-spectrum antimicrobials could substantially reduce the time and effort required for auditing, thereby improving workflow continuity for feedback providers. Automation may facilitate more timely and direct feedback to prescribing physicians and support the guidance of appropriate empirical therapy through integration with electronic healthcare systems.^28^

Various facilitators and barriers may have influenced the impact of the ASP intervention in the participating hospitals. In hospital 1, the low activity of opinion leaders may have limited the potential effect. Nevertheless, half of the target audience attended the clinical lessons, and all provided feedback was incorporated. The ASP had a moderate effect only in hospital 2, despite already high baseline guideline adherence, which might be attributed to the highly active opinion leaders and a 52.6% e-learning completion rate. In hospital 3, the high intervention effect might be explained by short communication lines between physicians, combined with a high recommendation acceptance rate and nearly half of the target audience attending the clinical lessons. Finally, in hospital 4, both the influence of a nearby academic hospital and internal conflicting opinions may have hindered the intervention, as reflected in a low feedback acceptance rate of 41.8%.

A limitation of this study is that not all data necessary for a full process evaluation were collected. Specifically, the exact time required to prepare clinical lessons, exposure to follow-up lessons, use of pocket cards and poster, and the actual performance of local opinion leaders were not recorded. Nevertheless, the available data provide sufficient detail to understand the caveats and key drivers of a successful ASP and facilitate its implementation in other settings. Although interviews were conducted two years after the trial, all participating hospitals were actively using intervention components at the time, which may have minimized the risk of recall bias. Only four hospitals were interviewed and inclusion of all hospitals might have provided additional contextual insights. However, saturation was reached after eleven interviews and similar components were perceived as most effective, irrespective of hospital-level outcomes. Therefore, these data provide a comprehensive overview of the perceived component effectiveness.

To conclude, hospitals aiming to reduce broad-spectrum antimicrobial use in CAP patients may consider to conduct daily prospective audit and feedback, implement regular clinical lessons, distribute pocket cards, and appoint actively engaged local opinion leaders across relevant departments. Additionally, it is recommended to periodically report prescribing performance within the hospitals, compare performance between departments, and establish performance evaluation structures across hospitals.

## Supporting information

10.1017/ash.2026.10340.sm001 van Heijl et al. supplementary material van Heijl et al. supplementary material

## Supplementary material

The supplementary material for this article can be found at https://doi.org/10.1017/ash.2026.10340.

## Research transparency and reproducibility

The data set is available from the corresponding author on reasonable request.
