# Supplementary material for: Process evaluation of an effective Antimicrobial Stewardship intervention in hospitalized patients with community-acquired pneumonia
Source: Antimicrob Steward Healthc Epidemiol. 2026 Mar 30;6(1):e82. doi: 10.1017/ash.2026.10340 (PMC13104519; doi:10.1017/ash.2026.10340)
Supplement: van Heijl et al. supplementary material [file S2732494X26103404sup001.docx]

Supplement to:

**Process evaluation of an effective antimicrobial stewardship intervention in hospitalized patients with community-acquired pneumonia**

**Table of contents**

[S1. Antimicrobial stewardship intervention bundle – First clinical lesson 2](#_Toc216445155)

[S2. Antimicrobial stewardship intervention bundle – E-learning 9](#_Toc216445156)

[S3. Antimicrobial stewardship intervention bundle – Pocket card 10](#_Toc216445157)

[S4. Antimicrobial stewardship intervention bundle – Poster 11](#_Toc216445158)

[S5. Antimicrobial stewardship intervention bundle – Prospective Audit and Feedback 12](#_Toc216445159)

[S6. Effective Practice and Organization of Care (EPOC) Taxonomy 13](#_Toc216445160)

[S7. Themes in interview guide 14](#_Toc216445161)

[S8. Prospective audit and feedback results 15](#_Toc216445162)

[S9. Clinical lesson feedback form 17](#_Toc216445163)

[S10. Results clinical lesson feedback forms 18](#_Toc216445164)

[References 19](#_Toc216445165)

# S1. Antimicrobial stewardship intervention bundle – First clinical lesson


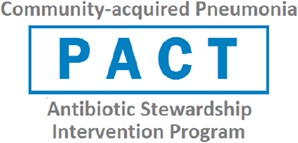


Logo of

participating hospital

“presenter”


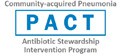


Content

- Introduction
- SWAB CAP guideline and questions
- Results; how is a CAP treated in clinical practice?
- Intervention


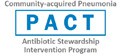


Introduction

Community-acquired pneumonia

Pneumonia acquired outside the hospital

Hospital-acquired pneumonia

Pneumonia >48hr after hospital admission


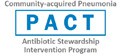


SWAB CAP guideline and questions


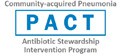

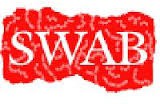


SWAB CAP guideline (question)

*A 67-year-old man is admitted to the Pulmonology department with a working diagnosis of CAP. To start the appropriate empirical treatment, you first determine the disease-severity. You can choose from one of the three scoring systems; PSI score, CURB-65 score or pragmatic classification.*

Is there a preferred scoring system according to the guideline to determine disease-severity of CAP?

No

1. SWAB CAP guideline version 2011


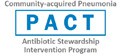

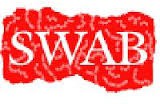


SWAB CAP guideline (question)

*A 67-year-old man is admitted to the Pulmonology department with a working diagnosis of CAP. To start the appropriate empirical treatment, you first determine the disease-severity. You can choose from one of the three scoring systems; PSI score, CURB-65 score or pragmatic classification.*

Is there a preferred scoring system according to the guideline to determine disease-severity of CAP?

1. SWAB CAP guideline version 2011


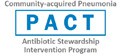


Introduction

- Empirical treatment is based on disease-severity
- Three different scoring systems

– SWAB CAP guideline: no preference

|  | Classification | | |
| --- | --- | --- | --- |
| Disease-severity CURB-65 | | Pragmatic | PSI |
| Mild CAP 0-1 | | Treatment at home | 0-1 |
| Moderate-severe CAP 2 | | Admission to a general medical ward | 2 |
| Severe CAP 3-5 | | Admission to an ICU | 3-5 |


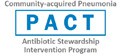

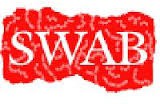


SWAB CAP guideline (question)

*According to the pragmatic classification the CAP of the 67-year-old man is classified as moderate-severe because he is admitted to a general medical ward.*

Which empirical treatment is recommended for this patient?

1. SWAB CAP guideline version 2011


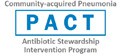

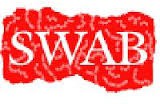


SWAB CAP guideline (question)

*According to the pragmatic classification the CAP of the 67-year-old man is classified as moderate-severe because he is admitted to a general medical ward.*

Which empirical treatment is recommended for this patient?

Amoxicillin / benzylpenicillin

1. SWAB CAP guideline version 2011


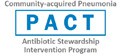

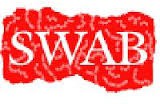

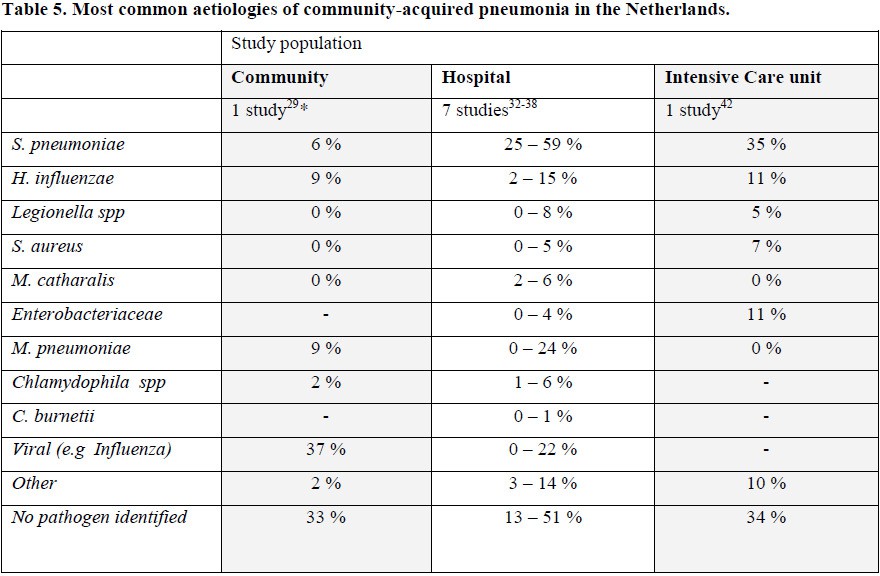


SWAB CAP guideline

1. SWAB CAP guideline version 2011


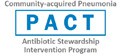

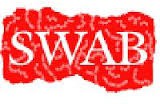


SWAB CAP guideline (question)

What is the prevalence of penicillin-resistant *Streptococcus pneumoniae*

in the Netherlands?

1. SWAB CAP guideline version 2011


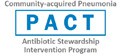

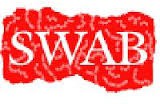


SWAB CAP guideline (question)

What is the prevalence of penicillin-resistant *Streptococcus pneumoniae*

in the Netherlands?

<1%

1. SWAB CAP guideline version 2011


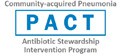

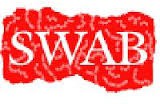

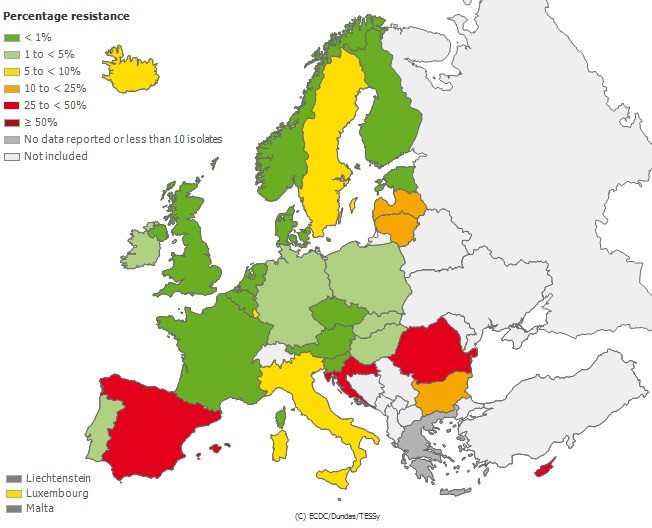


SWAB CAP guideline

1. Antimicrobial resistance interactive database (EARS-Net) 2012


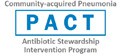

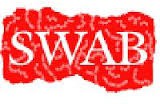


SWAB: CAP guideline (question)

*Prior to the admission at the Pulmonology department a colleague at the Emergency Room already administered one dose of ceftriaxone to the 67- year-old man. Therefore, before treatment de-escalation, you first perform the pneumococcal urine antigen test. What is the positive predictive value of this test?*

What is the positive predictive value of this test?

1. SWAB CAP guideline version 2011


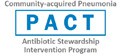

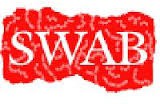


SWAB: CAP guideline (question)

*Prior to the admission at the Pulmonology department a colleague at the Emergency Room already administered one dose of ceftriaxone to the 67- year-old man. Therefore, before treatment de-escalation, you first perform the pneumococcal urine antigen test. What is the positive predictive value of this test?*

What is the positive predictive value of this test?

89-97%

1. SWAB CAP guideline version 2011


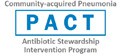

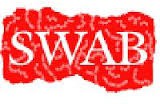


SWAB CAP guideline (question)

*The turnaround time of a pneumococcal urine antigen test is 15 minutes. One hour after you performed this test, you phone the Microbiology department. The test result is negative. In the meantime, your 67-year-old patient does not improve after 1 day of treatment with ceftriaxone. You consider adding ciprofloxacin to the initial therapy to cover a possible Legionella infection.*

In which situation is it appropriate to add antibiotic treatment for *Legionella*

infection?

1. SWAB CAP guideline version 2011


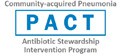

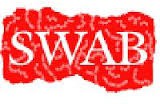


SWAB CAP guideline (question)

*The turnaround time of a pneumococcal urine antigen test is 15 minutes. One hour after you performed this test, you phone the Microbiology department. The test result is negative. In the meantime, your 67-year-old patient does not improve after 1 day of treatment with ceftriaxone. You consider adding ciprofloxacin to the initial therapy to cover a possible Legionella infection.*

In which situation is it appropriate to add antibiotic treatment for *Legionella*

infection?

Failure to improve despite ≥ 48 hours treatment with a beta-lactam antibiotic at adequate dosage.

1. SWAB CAP guideline version 2011


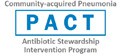

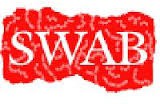

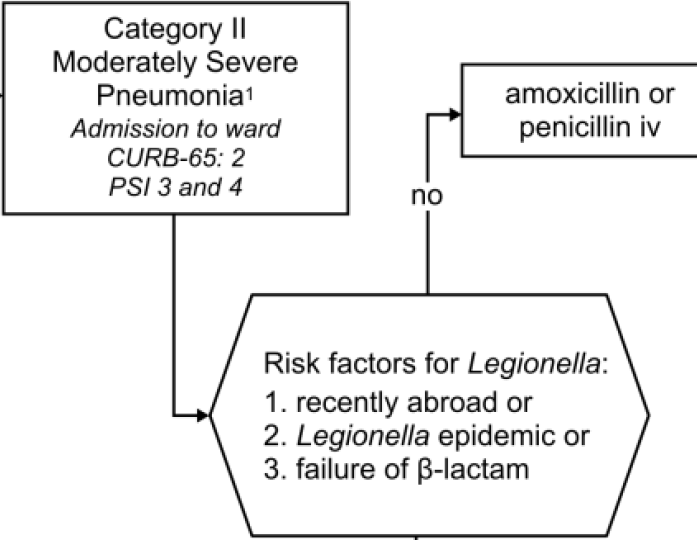


SWAB CAP guideline

1. SWAB CAP guideline version 2011


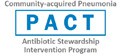

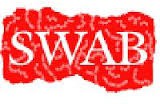

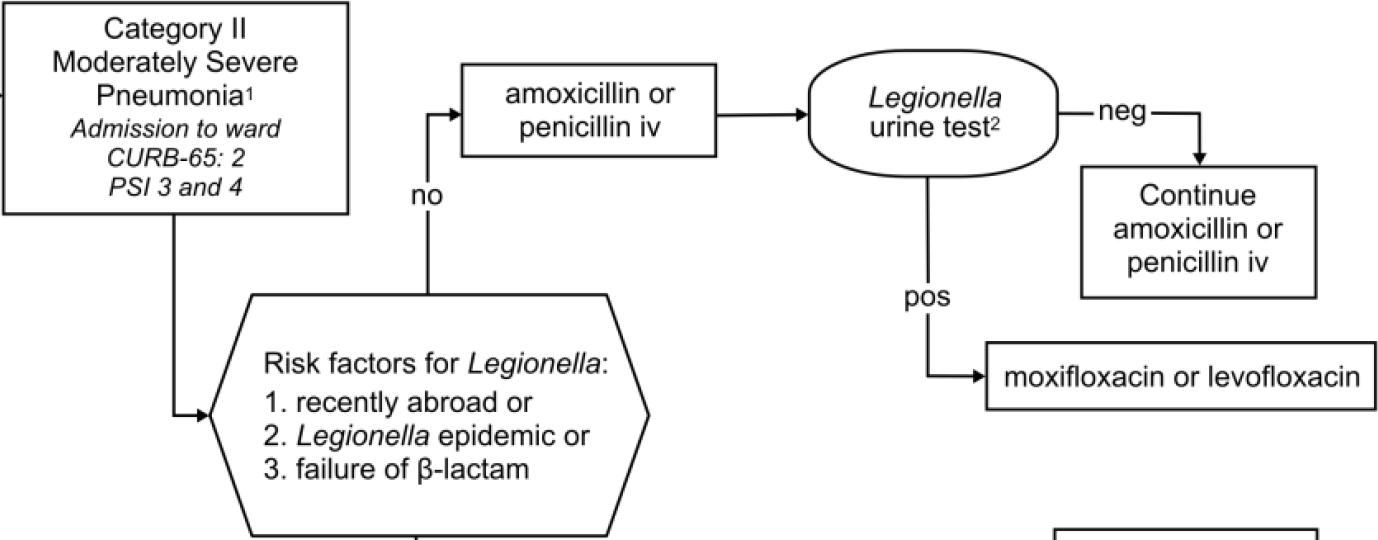


SWAB CAP guideline

1. SWAB CAP guideline version 2011

Ciprofloxacine


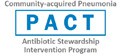

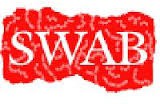

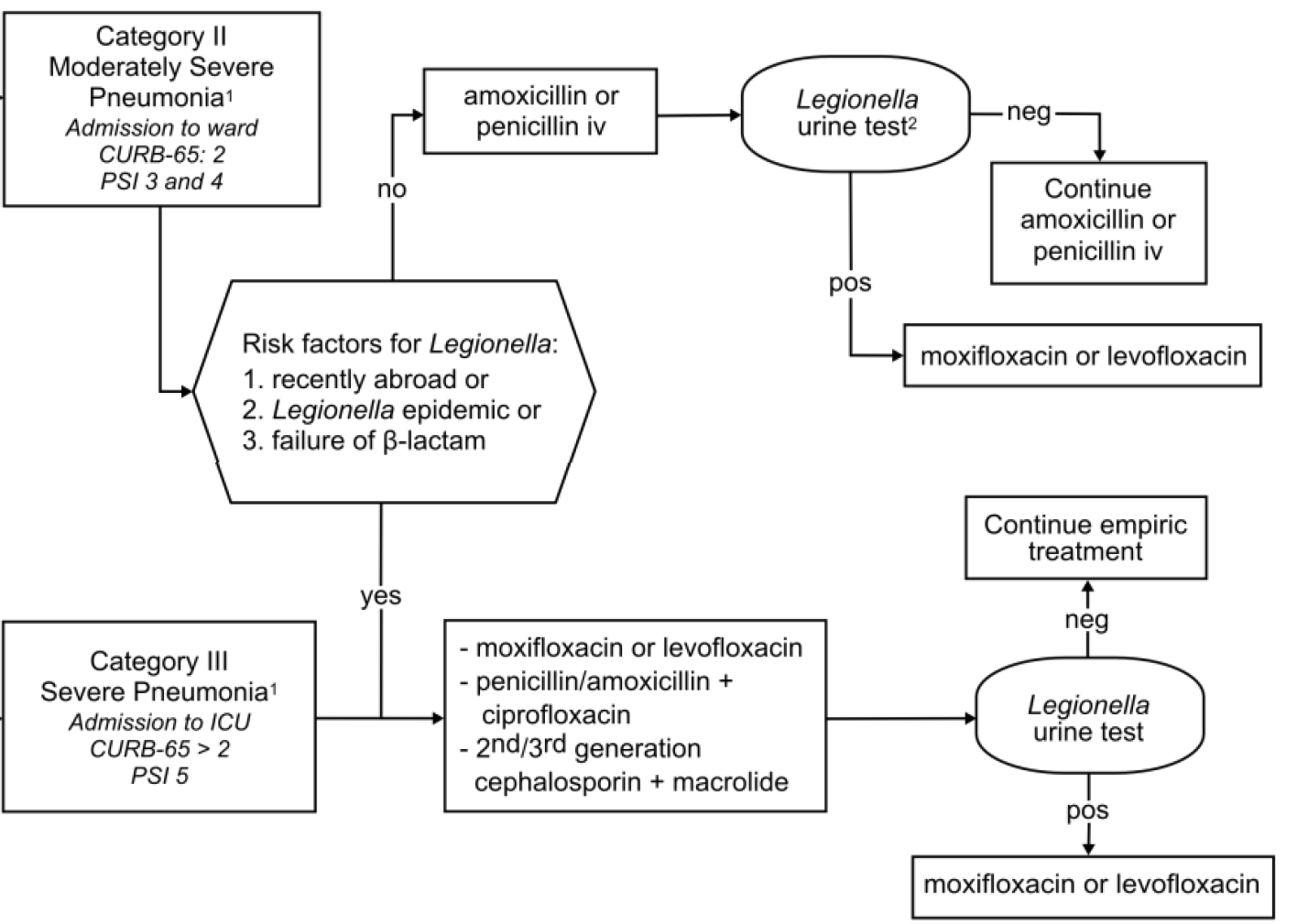


SWAB CAP guideline

1. SWAB CAP guideline version 2011

Ciprofloxacine


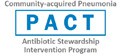

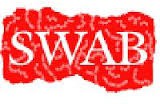


SWAB CAP guideline (question)

What is the chance to identify Legionella as a causative pathogen in patients with non-severe CAP?

1. Postma et al. NEJM 2015


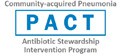

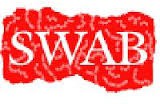


SWAB CAP guideline (question)

What is the chance to identify Legionella as a causative pathogen in patients with non-severe CAP?

0-1%

1. Postma et al. NEJM 2015


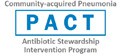

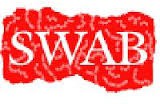

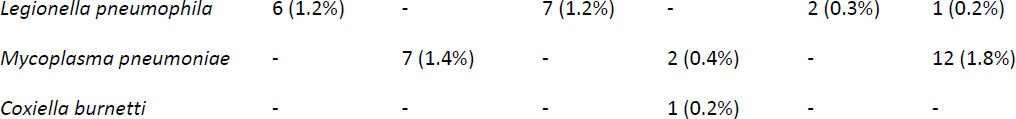

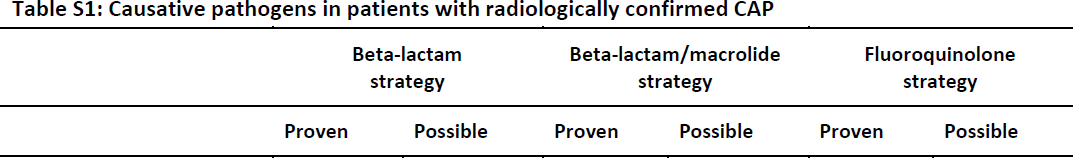


SWAB CAP guideline

1. Postma et al. NEJM 2015


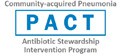

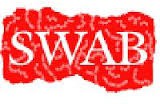


SWAB CAP guideline (question)

*During the medical handover a similar patient is presented by a colleague. However, this patient has a history of COPD.*

Is amoxicillin an appropriate empirical antibiotic therapy for a COPD patient with a moderate-severe CAP?

1. SWAB CAP guideline version 2011


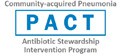


Results of control period

100

90

80

70

60

50

40

30

20

10

0

Internal medicine

Pulmonology

**% patients**


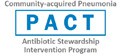

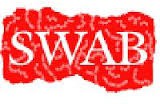


SWAB CAP guideline (question)

*During the medical handover a similar patient is presented by a colleague. However, this patient has a history of COPD.*

Is amoxicillin an appropriate empirical antibiotic therapy for a COPD patient with a moderate-severe CAP?

Yes

1. SWAB CAP guideline version 2011


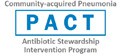

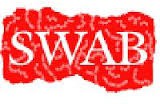

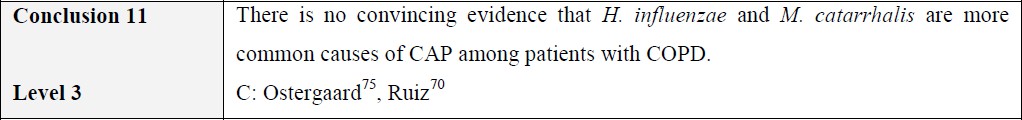


SWAB CAP guideline

1. SWAB CAP guideline version 2011


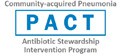


How is a CAP treated in clinical practice?

Results of control period


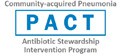


Results of control period

- Number of patients included: X
  - Patients with working diagnosis of CAP
  - Period: November 2015 till XX.XX.XX


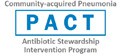


Results of control period

Patient characteristics

Age (± SD)

Gender (male, %)

Nursing home resident (n, %)

Smoking behavior (n, %)

Yes No

Unknown

*n* = X

X ± X

X% X%

X%

X% X%


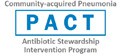


Results of control period

Co-morbidities

*n* = X


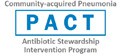


Results of control period


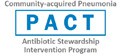


Results of control period

100

PUAT LUAT

90

80

70

60

50

40

30

20

10

0

A

C

D

E

F

G

I

J

% patients

| Heart failure | X% |
| --- | --- |
| Cerebrovascular disease | X% |
| Malignancy | X% |
| Chronic liver disease | X% |
| Chronic renal disease | X% |
| Diabetes mellitus | X% |
| Asthma/COPD | X% |

| Antibiotic use before admission | *n* = X |
| --- | --- |
| Amoxicillin | X% |
| Amoxicillin/clavulanate | X% |
| Azithromycin | X% |
| Ciprofloxacin | X% |
| Doxycycline | X% |
| No antibiotics | X% |


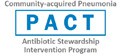


Results of control period

Diagnostics

Sputum culture X%

Blood culture X%

Pneumococcal urine antigen test X% Legionella urine antigen test X%


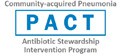


Results of control period

Empirical therapy during admission

Amoxicillin Amoxicillin/clavulanate

Amoxicillin/clavulanate + ciprofloxacin

Benzylpenicillin or amoxicillin + ciprofloxacin

Ceftriaxone Cefuroxime

Other

*n* = X

X%

X% X%

X%

X%

X%


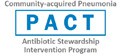


Intervention period


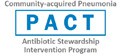


Follow-up: intervention period

- Pocket cards, posters.
- E-learning
- Daily screening and feedback from A-team


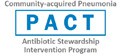


Questions?

- Researchers of CAP-PACT study:
  - Inger van Heijl
  - Valentijn Schweitzer
- A-team members “hospital”:
  - XXXX
  - XXXX
  - XXXX

**Figure S1. Example of a clinical lesson**

# S2. Antimicrobial stewardship intervention bundle – E-learning

**E-LEARNING COMMUNITY-ACQUIRED PNEUMONIA**


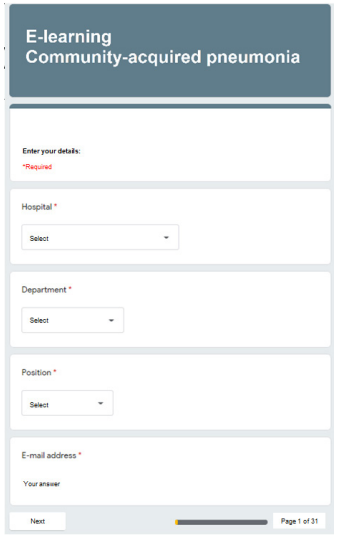


**Figure S2. Screenshot of the e-learning**

This e-learning was made via Google Forms.

**Hospital:** Dropdown menu with names of participating hospitals.

**Department:** Dropdown menu with: Internal Medicine, Pulmonology, Emergency room, Geriatrics,

Medical Microbiology.

**Position:** Dropdown menu with: Medical specialist, Resident.

**E-mail address:** [Open text field]

**Welcome:** Welcome to the e-learning for the treatment of hospitalized patients with community-acquired pneumonia (CAP). This e-learning is an initiative from the Antimicrobial

Stewardship team (A-team) to improve the treatment of hospitalized patients with CAP

in your hospital.

**Aim:** The aim of this e-learning is to gain inside into the treatment of CAP.

**Content:** De e-learning consists of a short introduction following a number of multiple-choice questions. You can consult the corresponding literature at any time while answering the questions. The guideline on community-acquired pneumonia is available at http://www.swab.nl/richtlijnen. After you have given your answer, you directly see whether this was correct. You will find the references of the questions at the end of the e-learning.

**Time:** The total amount of time to complete the e-learning will be approximately 10 minutes.

# S3. Antimicrobial stewardship intervention bundle – Pocket card

**CAP-PACT study**
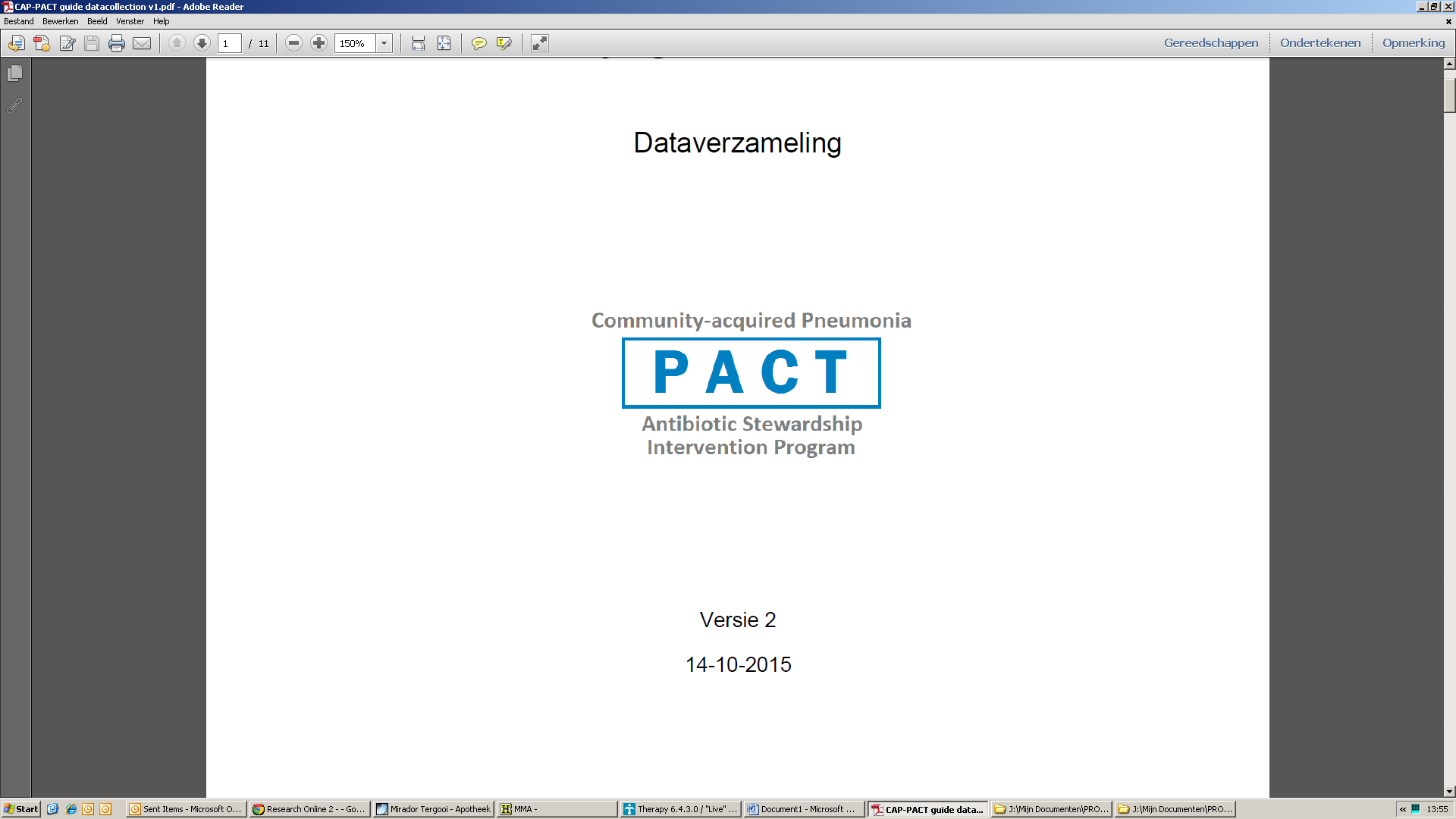


**Community-acquired pneumonia**

**Pneumonia acquired outside the hospital**

- No recent hospital admission >48hr (<2 weeks)
- No recent residence in long-term care facility >48hr (<2 weeks)

**Classification**

| **Disease severity** | **Classification** |  |  |
| --- | --- | --- | --- |
|  | *CURB-65* | *Pragmatic score* | *PSI score* |
| Mild CAP | 0-1 | No admission | 1-2 |
| Moderate-severe CAP | 2 | Admission at a non-ICU ward | 3-4 |
| Severe CAP | 3-5 | ICU admission | 5 |

**Diagnostics**

Blood culture, sputum culture and pneumococcal urinary antigen test

Legionella urinary antigen test only in patients with a severe CAP or with risk factors for Legionnaires disease. Risk factors:

- Travel abroad
- Legionnaires disease outbreak
- No effect of penicillins or cephalosporins after 48 hours

**Empirical therapy**

| **Disease severity** | **Antibiotics** | **Route** |
| --- | --- | --- |
| *Mild CAP* |  |  |
| 1^st^ line | Amoxicillin | Oral |
| 2^nd^ line | Doxycycline | Oral |
| *Moderate-severe* *CAP* |  |  |
| 1^st^ line | Benzylpenicillin or  Amoxicillin | IV/oral |
| *Severe CAP* |  |  |
| Combination therapy | Benzylpenicillin + ciprofloxacin | IV |

[HOSPITAL LOGO]

**Figure S3. Example of a pocket card**

# S4. Antimicrobial stewardship intervention bundle – Poster


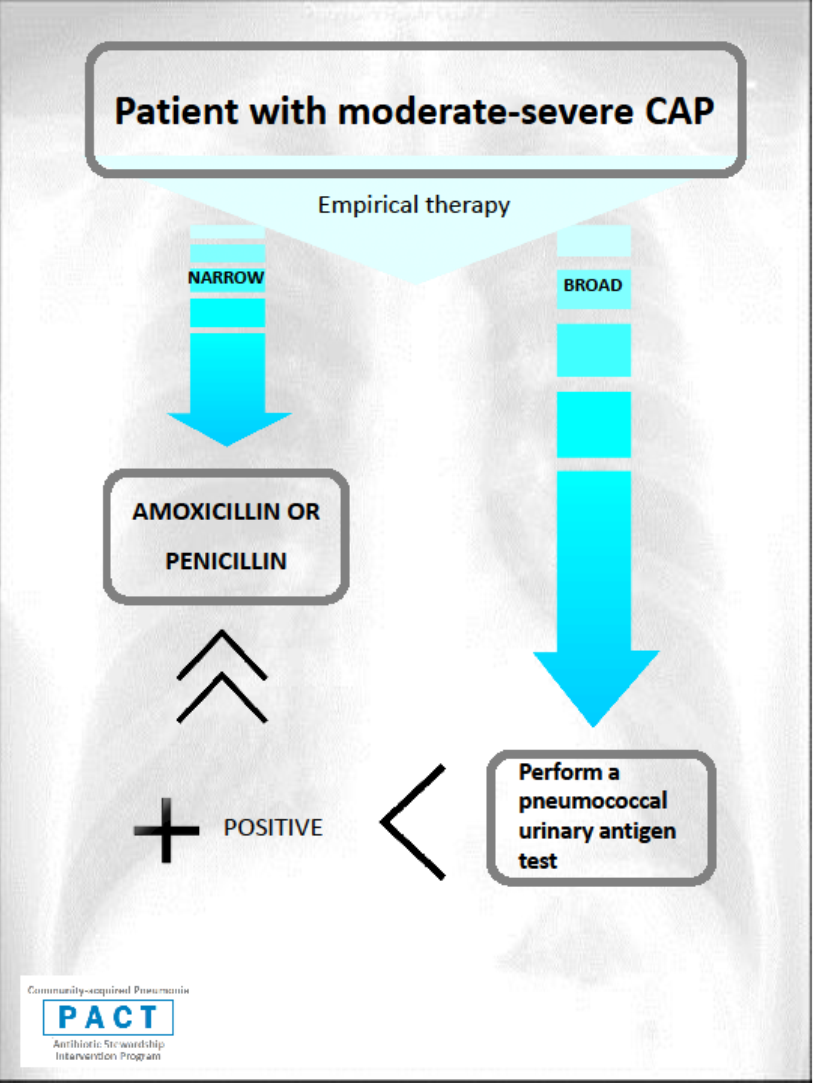


**Figure S4. Example of the poster**

# S5. Antimicrobial stewardship intervention bundle – Prospective Audit and Feedback

**Table S5. Personnel responsible for conducting prospective audit and feedback**

| **Hospital** | **Prospective audit and feedback conducted by** |
| --- | --- |
| 1 | Audit: research nurse of the Pulmonology department.  Feedback: microbiologist resident responsible for daily shift. |
| 2 | Audit: hospital pharmacist*.  Feedback: microbiologist responsible for daily shift. |
| 3 | Audit: microbiologist* responsible for daily shift.  Feedback: microbiologist* responsible for daily shift. |
| 4 | Audit: research nurse of the Microbiology department.  Feedback: microbiologist resident responsible for daily shift. |
| 5 | Audit: pulmonologist*.  Feedback: hospital pharmacist responsible for daily shift. |
| 6 | Audit: hospital pharmacist*.  Feedback: hospital pharmacist*. |
| 7 | Audit: research nurse* of the Microbiology department.  Feedback: research nurse* of the Microbiology department. |
| 8 | Audit: research physician* of the Microbiology department.  Feedback: research physician* of the Microbiology department. |
| 9 | Audit: research nurse* of the Clinical Pharmacy department.  Feedback: research nurse* of the Clinical Pharmacy department. |

* These persons were part of the local antimicrobial stewardship teams.

# S6. Effective Practice and Organization of Care (EPOC) Taxonomy

**Table S6. Intervention components of ASP strategy according to EPOC taxonomy.^1^**

| Intervention components | EPOC subcategory |
| --- | --- |
| (A) Educational activities |  |
| Clinical lessons | Educational meetings |
| Feedback aggregated antibiotic use (integrated in clinical lessons) | Monitoring the performance of the delivery of healthcare |
| E-learning | Educational materials |
| Pocket card | Reminders |
| Poster | Reminders |
| (B) Engaging opinion leaders |  |
| One opinion leader per hospital per relevant specialty | Local opinion leaders |
| (C) Prospective audit and feedback of antibiotic use |  |
| Audit of antibiotic therapy in hospitalized CAP patients, with feedback from the antimicrobial stewardship team members | Audit and feedback |

# S7. Themes in interview guide

**Table S7. Themes used in interview guide**

| Key features | Themes (experiences and suggestions for improvement) |
| --- | --- |
| Target group | Appropriateness of the target group  Barriers and facilitators for participation |
| Implementer | Appropriateness of presenting professionals |
| Intensity | Frequency, duration, timing |
| Information imparted | Quality of content, presentation form, type of information, medium |
| Information about target group performance | Quality of content, presentation form, type of information, medium |

# S8. Prospective audit and feedback results

**Table S8. Prospective audit and feedback results**

|  | Intervention period (n=1,849) |
| --- | --- |
| Patients eligible for feedback | 591 (32.0 %) |
| Recommendations given | 330 / 591 (55.8%) |
| Accepted | 197 / 330 (59.7%) |
| Rejected | 133 / 330 (40.3%) |
| Reasons for rejection |  |
| Antibiotics discontinued | 5 (3.8 %) |
| Patient discharged or deceased | 4 (3.0%) |
| Penicillin allergy | 2 (1.5 %) |
| Legionella risk factors | 2 (1.5 %) |
| Advice of microbiologist | 1 (0.8%) |
| Severe pneumonia by PSI/CURB score / clinical deterioration | 9 (6.8%) |
| COPD* | 11 (8.3%) |
| Suspected resistant pathogen | 9 (6.8%) |
| Treatment based on resistant pathogen in new culture | 6 (4.5%) |
| Treatment based on resistant pathogen in old culture | 2 (1.5 %) |
| Pneumococcal urine antigen test is negative | 8 (6.0%) |
| Pneumococcal urine antigen test forgotten | 5 (3.8%) |
| Supervisor wants to continue antibiotics | 7 (5.3%) |
| Reason not clear | 52 (39.0%) |
| Other reasons^1^ | 10 (7.5%) |
| Recommendations given by |  |
| Telephone | 244 (74.0%) |
| Medical record | 17 (5.1%) |
| Both | 69 (20.9%) |
| No recommendations given | 261 (44.2%) |
| Reasons for no recommendation given |  |
| Severe pneumonia based on PSI/CURB score or clinical deterioration | 56 (21.5%) |
| COPD* | 12 (4.6%) |
| Suspected resistant pathogen | 21 (8.0%) |
| Treatment based on resistant pathogen in old culture | 39 (14.9%) |
| No time to give advice / missed | 42 (16.1%) |
| Reason not clear | 28 (10.7%) |
| Other reasons^2^ | 63 (24.1%) |
| Patients not eligible for feedback | 1258 (64.0%) |
| Reasons for non-eligibility |  |
| Started with narrow-spectrum antibiotics | 866 (68.8%) |
| Switched to narrow-spectrum antibiotics | 89 (7.1%) |
| Antibiotics discontinued | 11 (0.9%) |
| Patient discharged or deceased | 31 (2.5%) |
| Penicillin allergy | 125 (9.9%) |
| Legionella risk factors | 107 (8.5%) |
| Advice of microbiologist | 29 (2.3%) |
| Second recommendations given | 13 (3.9%) |
| Accepted | 3 (0.9%) |
| Rejected | 10 (3.0%) |
| Second recommendations given by |  |
| Telephone | 6 (46.2%) |
| Medical record | 5 (38.5%) |
| Both | 2 (15.4%) |

* Chronic Obstructive Pulmonary Disease,

1: Other reasons for rejecting advice; due to hospital-acquired pneumonia (not according to official risk factors), due to possible other focus, due to recurrent pneumonia, post-obstructive pneumonia, bronchiectasis or awaiting culture results.

2: Other reasons for no advice given: hospital-acquired pneumonia (not according to guideline risk factors), patient immunocompromised (not according to our definitions), suspected empyema, patient is agitated, possible other focus, awaiting culture results, post-obstructive pneumonia, due to legionella risk factors (not according to guideline risk factors), all cultures negative, possible abscess, continuing treatment of general practitioner.

# S9. Clinical lesson feedback form

**CAP-PACT study**
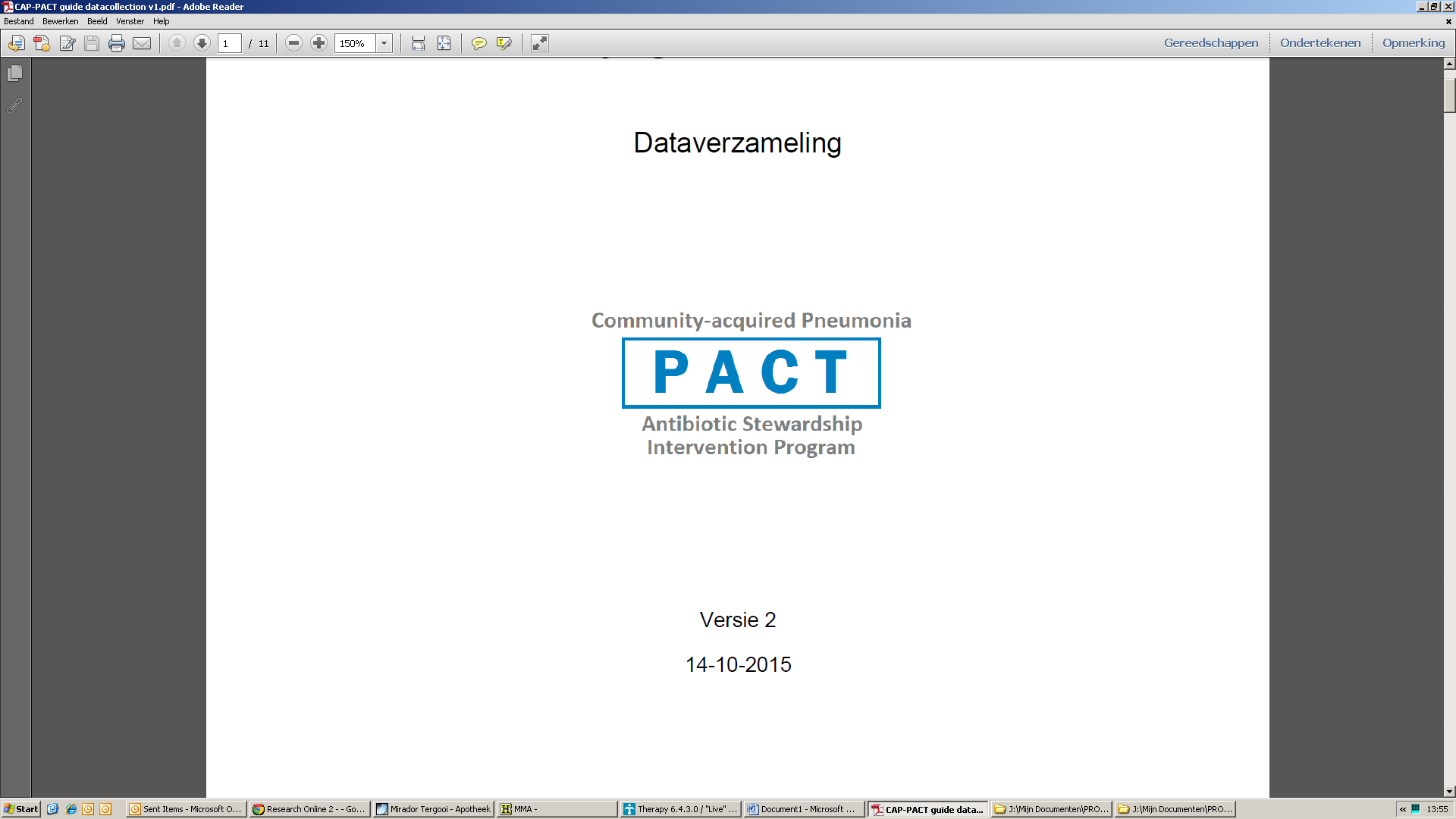


**Feedback form clinical lessons**

Date ………………………………………………………………………………………

**Content clinical lesson**

1 2 3 4 5 6

To what extent did this lesson meet your expectations?

To what extent did you acquire sufficient knowledge, insight and/or

skills with this lesson?

How do you rate the quality of the lesson?

How do you rate practical usability of the lesson?

|  |
| --- |
| **Speaker:** 1 2 3 4 5 6  How do you rate his/her form of presenting?  How do you rate the degree of interaction with speaker and participants?   \|  \|  \|  \|  \| \| --- \| --- \| --- \| --- \| \| Extra comments: \| \| \| \| |
|  |

| What did you like? |
| --- |
|  |
| What can be improved? |
|  |

[1 = bad; 2 = insufficient; 3 = moderate; 4 = sufficient: 5 = good; 6 = excellent]

**Figure S9. Clinical lesson feedback form**

# S10. Results clinical lesson feedback forms

**Table S10. Result feedback forms after the first two clinical lessons, aggregated over seven hospitals^a^**

| Question | Result (mean ± standard deviation) |
| --- | --- |
| To what extent did this lesson meet your expectations? | 4.3 ± 0.7 |
| To what extent did you acquire sufficient knowledge, insight and/or skills with this lesson? | 4.1 ± 0.9 |
| How do you rate the quality of the lesson? | 4.4 ± 0.7 |
| How do you rate practical usability of the lesson? | 4.5 ± 0.9 |
| How do you rate his/her form of presenting? | 4.7 ± 0.6 |
| How do you rate the degree of interaction with speaker and participants? | 4.8 ± 0.7 |
| What did you like? | Useful, practical, clear, interesting, short duration, structure, case-based discussion, presented results and degree of interaction with the public |
| What can be improved? | Aim was not clear, too much repetition of the content, change open-ended case-based questions to multiple-choice questions, increase difficulty level (especially for advanced residents and specialists), add more interaction, add more case-based questions and shorten the duration |

a. Feedback forms from two hospitals were unavailable as they were not distributed after the lessons.

# References

1. Effective Practice and Organisation of Care (EPOC). EPOC Taxonomy; 2015. Available from: epoc.cochrane.org/epoc-taxonomy Accessed July 3, 2025
